# The Use of SpineJack Intravertebral Implant for the Correction of Recurrent Vertebral Fracture After Kyphoplasty

**DOI:** 10.7759/cureus.7599

**Published:** 2020-04-09

**Authors:** Robert E Jacobson

**Affiliations:** 1 Neurological Surgery, University of Miami Hospital, Miami, USA

**Keywords:** kyphoplasty, spinejack, pmma, vertebral fractures

## Abstract

This report presents a case of short-term symptomatic failure with continued vertebral collapse after a T12 kyphoplasty for an acute fracture in a severely osteoporotic elderly patient. The original trajectory of the unilateral balloon and subsequently injected bone cement failed to fill the fracture, allowing further vertebral collapse that resulted in a rapid return of pain. Within 30 days, a titanium intravertebral body implant, SpineJack® (Stryker Corp, Kalamazoo, MI), combined with injection of polymethylmethacrylate (PMMA) bone cement, was placed in the collapsed area. This provided both sagittal and coronal partial correction of the collapse, fuller distribution of bone cement throughout the fractured vertebrae, and rapid reduction of pain. which was found to have been maintained at the long-term follow-up. The article reviews the technical issues causing failure of vertebral augmentation (VA) as well as the advantage of providing a permanent internal scaffolding to ensure stabilization of any fracture, especially where there is a high risk for progressive instability, such as the thoracic-lumbar junction.

## Introduction

Vertebral augmentation (VA) procedures include both vertebroplasty and kyphoplasty and, currently, third-generation implantable expanders all supplemented with bone cement. These procedures are frequently performed after failure of conservative treatment for osteoporotic compression fractures in patients, due to either persistent pain or progressing spinal deformity [1]. VA can be done either unilaterally or bilaterally, but results always depend on getting adequate cement placement and fill in the region of the fracture, which is most commonly under the superior endplate. In evaluating patients with persistent or recurrent pain after these procedures, the most common cause found is later development of adjacent-level compression fractures, usually above the treated level and, less commonly, recurrent fracture at the treated level [2-4]. Adjacent-level fractures are reported in up to 23% of VA procedures, statistically higher with kyphoplasty compared to vertebroplasty; recurrent fracture in the treated vertebra has been reported as early as seven days after an initial procedure in 3-11% of large series of cases, often in patients with untreated osteoporosis [3,5].

In the case reported here, there was concurrent severe osteoporosis, leading to a unilateral vertebral collapse of the untreated side. Patients that do get relief from the initial procedure or have a recurrence of pain within a very short time must be evaluated for the reasons for the failure of the actual procedure, such as continued collapse secondary to an inadequate amount of cement or absence of cement in the fractured area of the vertebrae, as well as the loss of the initial expansion after balloon kyphoplasty [6-8]. Specific technical reasons for recurrent pain and failure at the previously treated vertebral fracture include inadequate cement filling of the fracture region, such as the superior endplate and, especially, non-filling of an intravertebral cleft and, possibly, continued vertebral collapse from an evolving fracture at the interface between the cement and the osteoporotic bone [8,9]. Pre-existing intravertebral clefts and clefts developing after the procedure are one of the more overlooked radiologic findings of progressive collapse and can be accompanied by a gradual height decrease with the return of pain after an initially successful VA procedure. Contrast-enhanced MRI scans can help delineate such clefts in a high percentage of cases [10-12]. There is a clear role for repeat VA in previously treated vertebrae when there is a progression of the fracture and deformity, or if the patient develops recurrent pain [13,14]. The SpineJack® (Stryker Corp, Kalamazoo, MI) expandable intravertebral implant is a third-generation VA method that has been previously studied extensively for the treatment of primary osteoporotic fractures rather than for recurrent fractures [15].

## Case presentation

**Figure 1:**
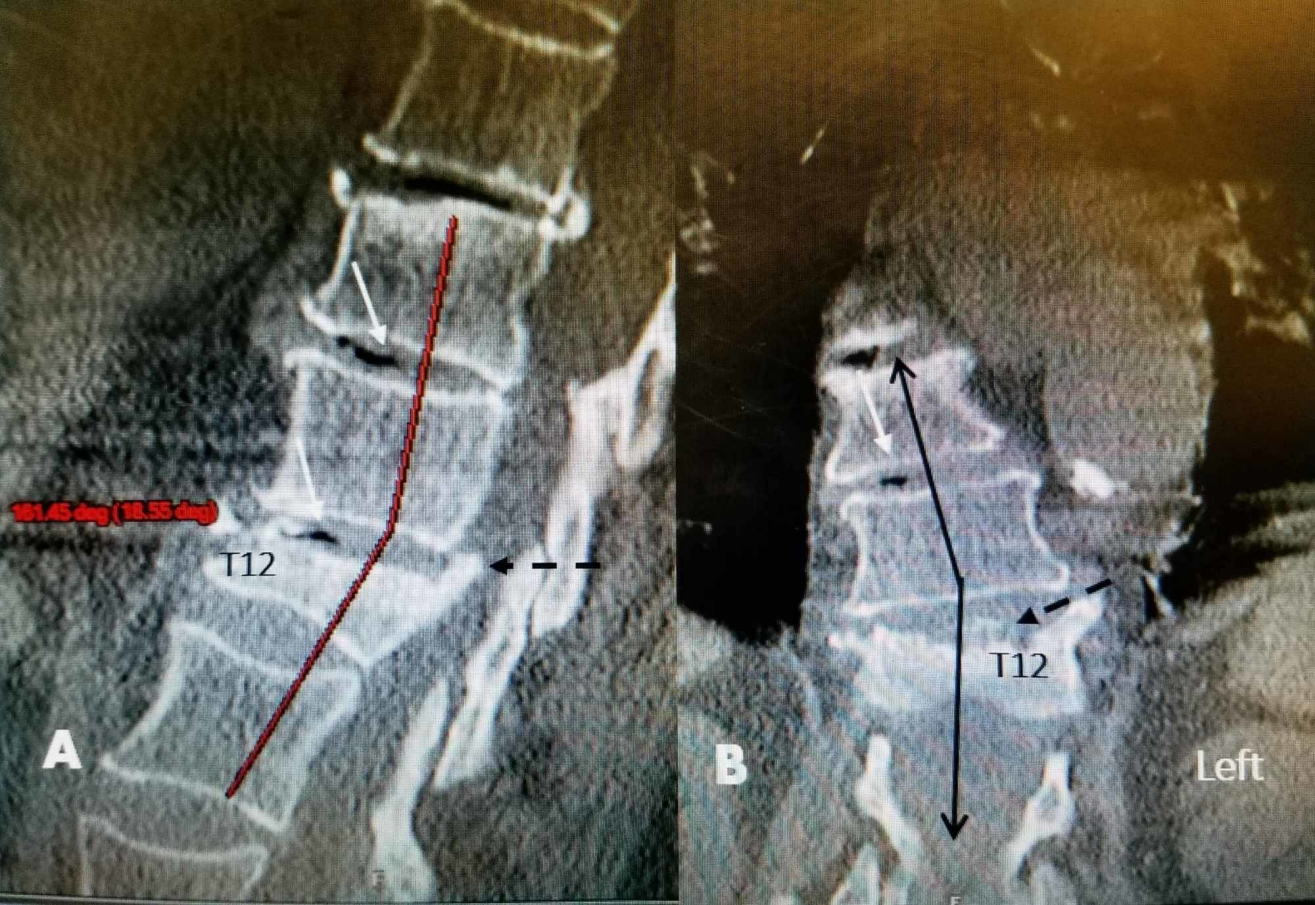
CT scan before initial kyphoplasty A: initial sagittal computerized tomography (CT) scan showing collapse of T12 with 10 degrees of kyphotic angulation (solid line) with dorsal displacement of the superior endplate of T12 into the spinal canal (dashed black arrow). B: coronal CT scan showing 10-degree angulation to the right of T11-T12. There is asymmetric collapse of the superior endplate of T12 more on the left side (dashed black arrow) CT: computed tomography

The patient was an 81-year-old female who had fell in her bathtub at home. She had severe pain and was initially seen by her primary care physician and placed in lumbar support. She had been on long-term clopidogrel 75 mg as an antiplatelet medication for chronic atrial fibrillation. She also had mild hypertension but had not been medically treated for osteoporosis. A bone mineral density (BMD) from six months prior to the fall had a reading of -4.3 for AP lumbar spine, -2.8 for femoral neck, and -1.8 for hip, indicative of severe osteoporosis. She underwent a CT of the lumbar spine almost six weeks after the fall, showing a 50% compression fracture of T12 with a 6-mm posterior displacement of the superior endplate of T12 into the canal. At that time, there were 10 degrees of spinal angulation to the right and five degrees of sagittal kyphosis at T12, spinal stenosis at L3-4 and L4-5 with partial auto-fusion of the disc space, and sagittal canal stenosis at L4-5 (Figure 1).

**Figure 2:**
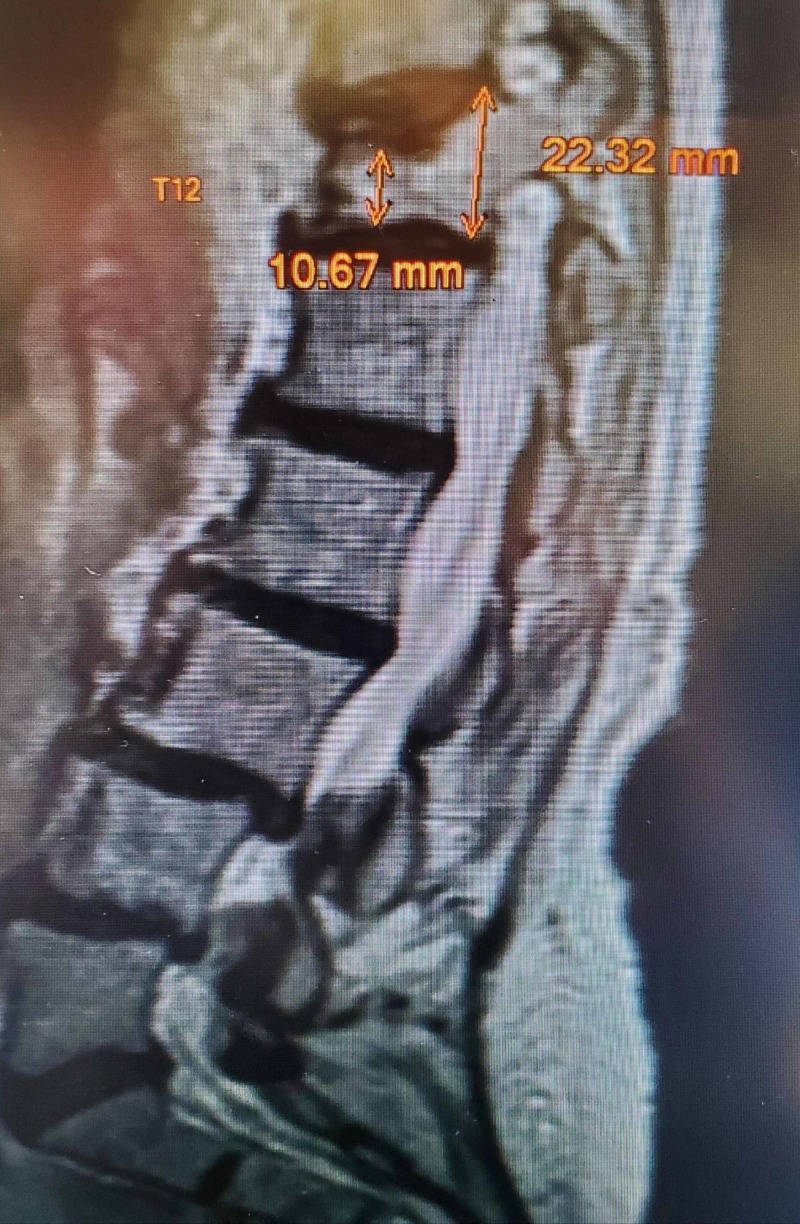
MRI obtained after persistent pain despite bracing Sagittal T2 MRI showing 60% anterior wedge collapse of T12 with lower spinal stenosis at L3-4 and L4-5 MRI: magnetic resonance imaging

She was sent to a neurosurgeon, to whom she stated that her pain had subsided after wearing the support, She then had an MRI, now 2.5 months after the fall, which showed the T12 fracture with edema of the superior 50% of T12 under the superior endplate of the T12 vertebral body. At that time, her visual analog scale (VAS) pain score was 10. She had a repeat MRI 3.5 months after the fall, showing a 53% collapse of T12 (Figure 2).

**Figure 3:**
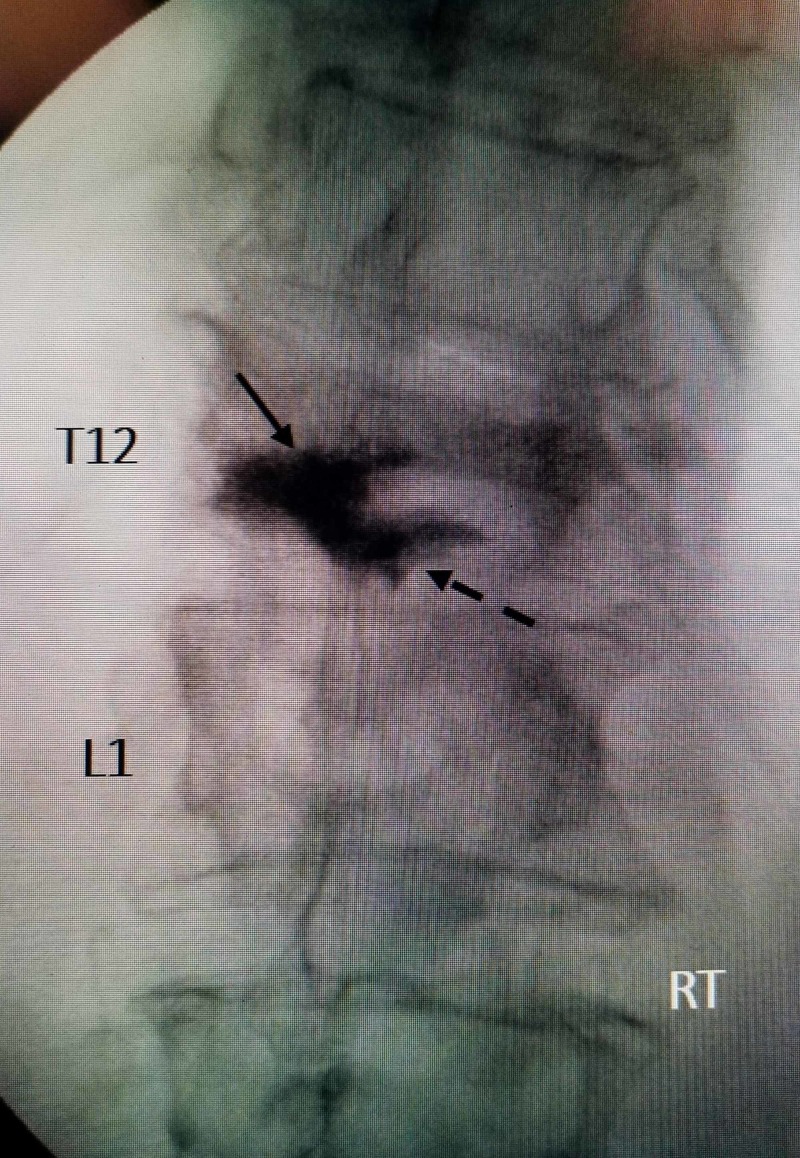
Postoperative film from initial balloon kyphoplasty The cement can be seen only on the left side inferiorly at T12 ( solid black arrow). There is spread of cement into the T12-L1 disc space inferiorly on the same side (dashed black arrow). The collapse and deformity to the right side is unchanged from pre-procedure films

Because of the persistent severe pain, she underwent a unilateral left balloon kyphoplasty. The surgeon's note stated that the pedicle bone was dense and it was difficult to place the balloon. Intra-operative films obtained afterwards showed the balloon cannula angled inferiorly through the right T12 pedicle, and postoperative plain radiographs showed that cement was seen mostly inferiorly and posteriorly on the right at T12, but there was also a spread of cement inferiorly into the T12-L1 disc space (Figure 3).

**Figure 4:**
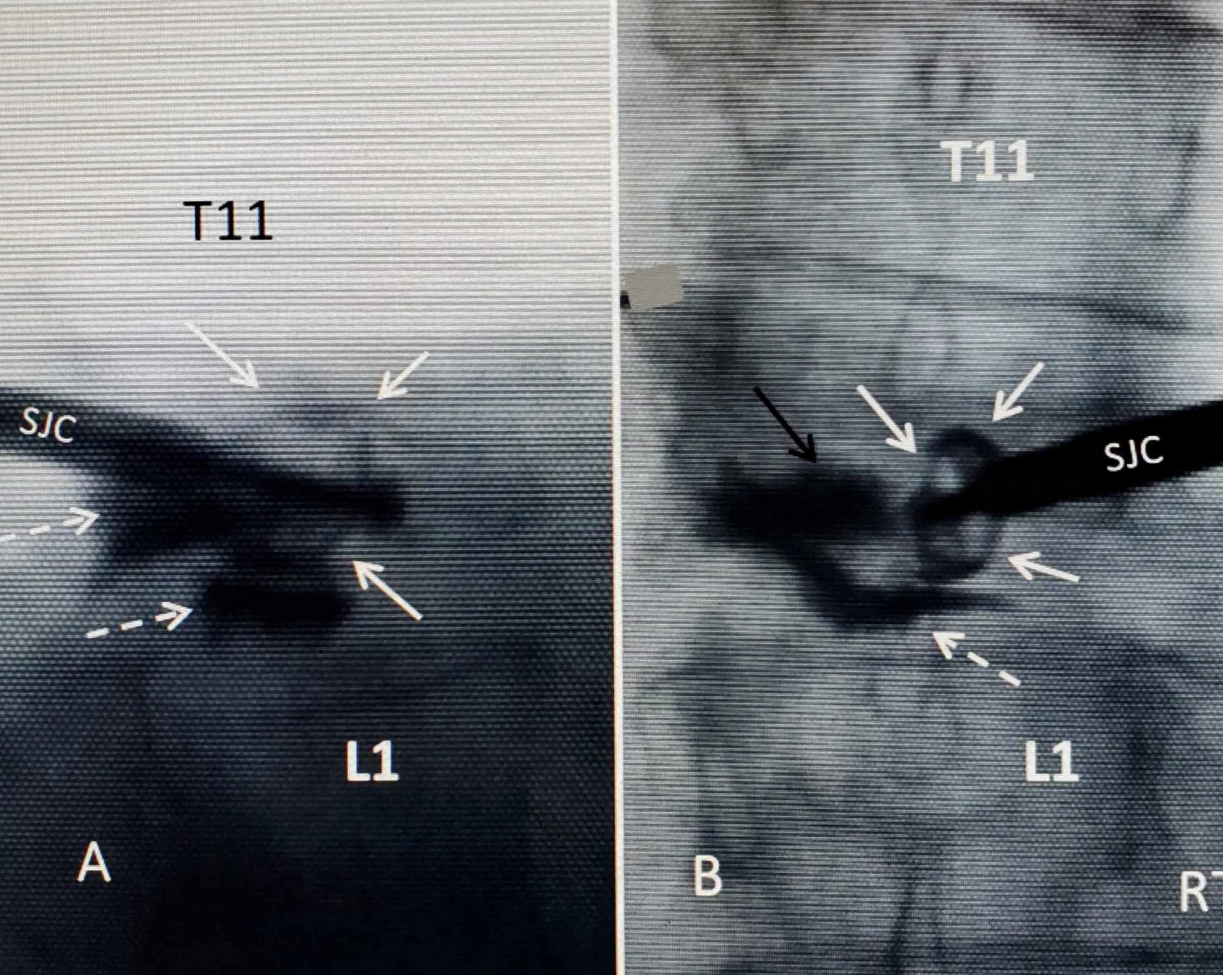
Intraoperative film of expanded 4.2-mm SpineJack implant just off-center from the right side of vertebral body before bone cement is injected A: lateral intra-operative film showing previously placed cement inferiorly and posteriorly (dashed white arrows). The SpineJack cannula (SJC) with attached implant is seen as well as the expanded wings (solid white arrows) of the implant superiorly and anterior to the previous balloon kyphoplasty. B: anterior-posterior intra-operative film. The patient's right side is marked, and the expanded implant attached to the SpineJack cannula (SJC) is seen to the right of the midline and previously left-sided kyphoplasty at T12 (solid white arrows). Cement is seen inferiorly within the T12-L1 disc space (dashed white arrow)

The patient was seen one week after the unilateral kyphoplasty procedure, and she stated that her pain had decreased to a VAS of two. However, two weeks later, and three weeks after the procedure, she was seen by a different neurosurgeon with complaints of worse pain, now back to a VAS of 10. Plain X-rays of her lumbar spine now showed an 18-degree thoracolumbar kyphosis, worsening collapse of T12 on the right, and cement fil only to left inferiorly at T12. It was recommended that the patient undergo repeat kyphoplasty at T12 on the right side. It was also recommended that a titanium SpineJack implant be inserted to restore height and to try and correct both the kyphosis and scoliosis. She underwent the procedure and a 4.2-mm SpineJack was placed from the right toward the midline (Figure 4).

**Figure 5:**
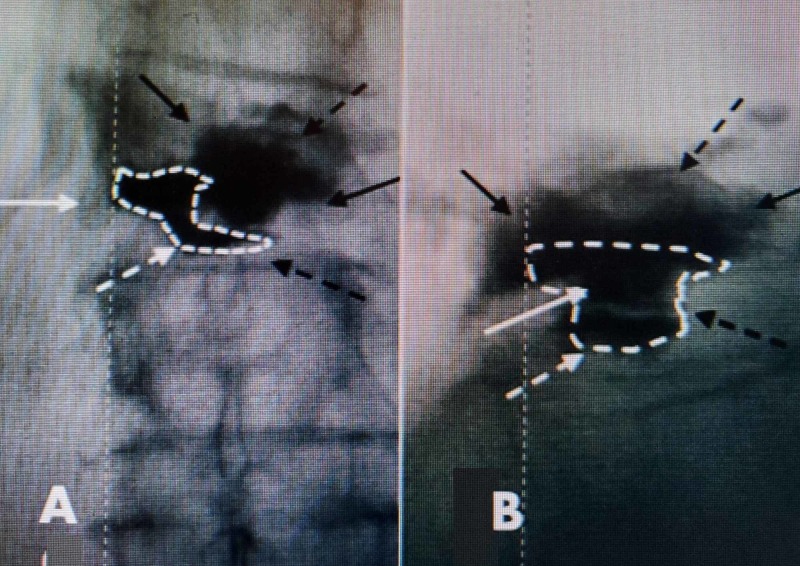
Post-injection intra-operative film showing comparison of pre- and post-injection cement pattern after 3.6 cc cement is injected through the SpineJack implant A: post-insertion anterior-posterior film after the insertion of a 4.2-mm SpineJack implant with an injection of PMMA. The implant was inserted so it was positioned in the midline. Previous pre-procedure cement is identified (dashed white line) while new cement fills across the midline to the opposite side and under the fracture of superior endplate. B: lateral image after insertion shows previous cement (dashed white outline) inferiorly and into the lower disc space compared to new PMMA cement fill from anterior to the posterior part of the fractured vertebrae especially under the superior endplate PMMA: polymethylmethacrylate

Comparison of pre- and post-insertion films of the implant and polymethylmethacrylate (PMMA) cement showed a much better central and bilateral fill pattern with 3.6 cc of PMMA cement with the spread of cement under the fractured superior endplate (Figure 5).

## Discussion

In reviewing the various factors in this case that contributed to the failure of the initial kyphoplasty resulting in continued collapse and severe pain, several salient points can be deduced. Clinically, the patient initially improved with the use of lumbar support; however, she returned within four weeks with severe pain despite using the support. Lumbar supports do not necessarily cover the thoracic-lumbar junction, and the patient's T12 fracture was associated with both kyphotic and scoliotic deformity [1,3,8]. Thoracolumbar fractures are known for their higher risk and instability [3,6,8]. The patient also had severe untreated osteoporosis with a BMD of -4.3 in the lumbar spine, markedly beyond the level of -2.5 for severe osteoporosis, and this indicated an increased risk of further collapse. There was also vacuum change in both the T10-T11 and T11-T12 disc spaces, and these changes and associated intravertebral clefts are often associated with an unstable fracture that may be more prone to progression despite VA [13].

There are several technical points regarding the original kyphoplasty procedure. The transpedicular approach was performed through the less severely collapsed side, and the more severely collapsed and fractured area of the T12 vertebra was never injected with cement. It would have been better to approach though the collapsed side first, or a cannula could have been placed and cement injected as vertebroplasty at the very least if an expanding balloon could not have been placed. The angle of approach also placed the balloon and cement too inferiorly, below the fractured endplate with significant leakage of cement into the disc space at T12-L1. Hence, the insufficient cement remained within the T12 fractured vertebrae, and as much as 30-40% of the injected cement is seen into the subjacent disc space at T11-T12 as seen in Figure 3.

More than 50% of fractures fill with a unilateral approach. However, in this case, when it was seen that both the angle of approach and cement location were inadequate and cement was neither directly under the fracture nor to the collapsed side, a balloon or simple vertebroplasty cannula should have been used on the opposite side, proceeding to a bilateral procedure [14]. Technically, when accessing the pedicle, it is also very important to align the trajectory of the cannula to the fractured endplate to ensure the proper distribution of cement. Recently, the introduction of third-generation systems such as the SpineJack, which places an expandable titanium implant under the fractured endplate acting as permanent internal support in osteoporotic fractures, has demonstrated a reduction in the percentage of recurrent adjacent-level fractures as well as better correction of underlying deformity [15]. Failure to place sufficient cement directly under the fractured endplate and, especially, within and fracture clefts, more commonly identified on MRI scans, is a common cause of recurrent collapses and procedure failures [16-18]. There are numerous reports that suggest that not getting adequate amounts of cement into the fracture area and not filling an intravertebral cleft are related to a higher rate of recurrent and adjacent-level fractures [10,13,16,17].

In treating recurrent fractures after failed kyphoplasty, it is absolutely necessary to make sure that cement gets into the proper region of the fractured vertebra and as much cement as possible is close to both endplates, especially under the fractured superior endplate (as in this case) [19]. Placing an expandable structural titanium intravertebral implant, rather than using a balloon in a patient with both significant collapse and previously placed PMMA cement, was the better option to minimize further collapse. In this case, a 4.2-mm implant was used, which expanded to 12.5 mm height x 20 mm length, and the addition of bone cement provided significantly larger volumetric support centered under the collapsed endplate bilaterally. Clinical experiences with the implant, especially in the treatment of primary thoracolumbar osteoporotic fractures at this level, supports this approach in failed kyphoplasty or recurrent fractures too [15,20].

## Conclusions

Treatment of osteoporotic fractures with different methods of VA is common and failures are infrequent. However, this case demonstrates how a combination of factors including severe osteoporosis, a fracture at the thoracic-lumbar junction, and insufficient cement in the correct location subjacent to the fractured endplate all contributed to rapid failure and recurrence of pain within several weeks. The SpineJack implant has been very effective in the treatment of primary vertebral fractures and has resulted in better height restoration and fewer adjacent-level fractures. The rationale for using a permanent intravertebral body SpineJack titanium implant is to provide a better means of permanent internal structural support in both the sagittal and coronal planes, achieve height restoration, and prevent further collapse of the fractured vertebrae. This case highlights its use for correcting recurrent fractures.
